# Developing Medical Geology in Uruguay: A Review

**DOI:** 10.3390/ijerph7051963

**Published:** 2010-04-28

**Authors:** Nelly Mañay

**Affiliations:** Toxicology & Environmental Hygiene Department, Faculty of Chemistry, University of the Republic of Uruguay, Gral, Flores 2124, 11800 Montevideo, Uruguay; E-Mail: nmanay@fq.edu.uy; Tel.: +598-2-9241-809; Fax: +598-2-9241-906

**Keywords:** Uruguay, medical geology, lead, arsenic, selenium, copper

## Abstract

Several disciplines like Environmental Toxicology, Epidemiology, Public Health and Geology have been the basis of the development of Medical Geology in Uruguay during the last decade. The knowledge and performance in environmental and health issues have been improved by joining similar aims research teams and experts from different institutions to face environmental problems dealing with the population’s exposure to metals and metalloids and their health impacts. Some of the Uruguayan Medical Geology examples are reviewed focusing on their multidisciplinary approach: Lead pollution and exposed children, selenium in critically ill patients, copper deficiency in cattle and arsenic risk assessment in ground water. Future actions are also presented.

## 1. Introduction

The emerging discipline of Medical Geology has been developed in Uruguay during the last decade linking Environmental Toxicology, Epidemiology, and Human and Veterinary health with Geology and Analytical studies, which have all contributed to scientific research in this field in Uruguay. The knowledge and application of these disciplines have been improved by joining research teams and experts from different institutions with community delegates to face environmental health problems arising from metal and metalloid exposure. Medical Geology has also been a matter of discussion in ecohealth symposiums to understand the relationships among ecosystems and human health impacts providing additional sociological studies.

The toxicology research team at the Faculty of Chemistry in Uruguay has been involved in this emerging discipline since 2002, studying metal and metalloid exposure and their environmental health impacts within the Medical Geology International group (IGCP project # 454) and the International Medical Geology Association (IMGA), created in 2005 [1]. Several contributions have been presented in scientific events, which were carried out locally and regionally, strengthening the integration of the biosciences, public health and geosciences with local communities and facilitating research and training opportunities among them.

## 2. Objectives

We present a review of these experiences in Uruguay, focusing on the development of a multidisciplinary and transdisciplinary approach. Available data concerning lead pollution as well as other research issues with regards to metals and metalloids like selenium, copper and arsenic are reviewed. We describe the Medical Geology field in Uruguay with some examples such as environmental lead exposure in children and selenium and copper deficiency in human and cattle populations and their related factors, in addition to recent studies on distribution, mobility and exposure to arsenic from ground water. These examples are described as follows:

### Lead pollution

The lead contamination in Uruguay, as in other countries in the region, is mainly due to its industrial sources (metallurgies, manufacture and recycling of batteries, foundries *etc*.). Generally, non-occupational lead exposure is caused by living in manufacturing areas or by the improper handling of lead-containing materials and solid waste, which poses an important health risk, with children being the most affected population [2]. Lead and other heavy metal exposure in Uruguayan populations has been studied at the Faculty of Chemistry for a long time with QA/QC analytical results [3]. Those scientific research publications turned out to be the only background data available for the whole country from when lead pollution first received official attention in 2001 [4–6], when the “La Teja neighborhood case” arose.

The first instance was a child from La Teja who showed blood lead levels (BLL) higher than 20 ug/dL, after which, several other cases of even higher values appeared in that neighborhood, as well as in other areas of Montevideo and throughout the whole country. The worst situation was in the slum settlements of La Teja, where the ground showed more than 3,000 ppm of lead in the soil due to scrap land fillings. This was later found in those areas because, foundries and metallurgical manufacturing industries had settled down during the last century, but most of them were no longer active due mainly to economic reasons, leaving their solid waste behind [5].

The community affected by lead contamination began a broad mobilization demanding solutions from the health and environmental authorities. As a response, the Health Ministry established an interinstitutional and multidisciplinary committee, including delegates from health, environmental, labor, educational, and social security institutions and community NGOs, among others. The University of the Republic was the main institution responsible for technical advice and support.

Lead pollution in Uruguay was then considered a Medical Geology issue, which highlighted the importance of the multidisciplinary, transdisciplinary and interinstitutional approach to provide solutions for the management of the health risks faced by thousands of affected children who were exposed to lead mainly due to the contaminated soil.

As a consequence of this approach, blood lead screening programs and lead protocols were implemented for Uruguayan children, specialized children health care centers were created and most blood lead levels determinations for health controls were confirmed at the Faculty of Chemistry. Moreover, many laws were approved, as well as several research projects which were being carried out [6].

The severity of the discovered lead pollution required official governmental actions, in order to reduce sources of lead contamination as well as to assess health impact on children who had been exposed to environmental or industrial lead pollution.

As those actions to reduce public exposure to lead, became consistent, lead studies performed by the toxicology team at the Faculty of Chemistry were reviewed to compare BLLs changes in Uruguayan populations, after the leaded gasoline was phased out in 2003 [7]. This report included comparisons of similar populations (children, exposed and unexposed adults, and lead workers) that were sampled within a 10-yr period. BLL determinations were all performed using atomic spectrometry at the Faculty of Chemistry using appropriate quality controls. The studies reviewed by the report, which were carried out in 2004, involved the sampling of children (*n* = 180), nonoccupationally exposed adults (*n* = 714), and lead workers (*n* = 81). A framework was established to correlate BLL with variables such as age, sex, area of residence, available environmental lead data, and possible lead exposure sources. To assess the change in risk, analytical results were statistically compared with similar screening studies results that were performed in 1994 by the same team.

Children in 1994 showed a positive relationship between BLL and traffic intensity, and 40% of their BLL exceeded 10 ug/dL, while in 2004, only 7% were above that intervention level. Results showed significantly lower BLL levels in children (5.7 ug/dL) and non occupationally exposed adults (5.5 ug/dL) than similar populations sampled in 1994 (9.9 ug/dL and 9.1 ug/dL, respectively). However, workers occupationally exposed to lead did not show significant BLL differences between 1994 and 2004, and their mean BLL values were still high (49.0 Pb ug/dL *vs*. 42.0 Pb ug/dL, respectively). In order to improve this, new laws have also been approved to address lead occupational exposure which required BLL assessment to be periodically monitored as part of the workers health certificate protocol.

It was shown that significant improvements in preventing nonoccupational lead exposure have been made and that this outcome can presumably be attributed to the phase-out of leaded gasoline and to improvements in nutrition, hygiene, and related habits for children as well as a favorable response to the official multidisciplinary actions. These changes suggest that the contribution of environmental lead to the overall exposure of children and non occupationally exposed adults in Uruguay is decreasing.

Recent studies have also demonstrated that there has been a significant change in preventing lead exposure due to the public’s sensitization, along with the integration of multidisciplinary actions promoted. As a conclusion, the problem of lead contamination in Uruguay has been considered an important and unique experience in Medical Geology development [6,7].

### Selenium deficiency and SIRS/MOD

A research study [8] was carried out by a multidisciplinary Medical Geology team including physicians, bioinorganic chemists and analytical toxicologists regarding Uruguayan serum selenium (Se) levels and glutathione peroxidase (GPx) activity in relation to oxidative stress such as systemic inflammatory response syndrome (SIRS) and multiple organ dysfunction (MOD).

Selenium status was evaluated in critically ill Uruguayan patients with and without SIRS in comparison with healthy subjects. Serum Se levels and GPx activity were measured in healthy subjects and patients without SIRS, with SIRS and with SIRS-MOD, who had been admitted to the Intensive Care Unit (ICU) in the Hospital de Clinicas of Montevideo. GPx activity was determined by an indirect method based on the oxidation of glutathione (GSH) to oxidized glutathione (GSSG), catalyzed by GPx, which was then coupled to the recycling of GSSG to GSH using glutathione reductase and NADPH. Se in serum was analyzed by graphite furnace atomic absorption spectrometry in our lab.

The results were expressed in mean values. As a preliminary conclusion, average Se levels in Uruguay (79 ug/L) seemed to be lower than in other populations, and these results have been confirmed by further research. SIRS is associated with Se depletion. For this reason, early Se supplementation could improve the results and prognosis in critically ill patients with SIRS [9].

### Copper deficiency in cattle

With regards to bovine health and geology, serum copper levels were studied in dairy cattle in eight locations throughout the Department of Salto, in Uruguay using a geomedical approach. The overall incidence of hypocupraemia was in 30%, which was not homogeneously distributed since some farms were almost exempt from hypocupraemical individuals. One of the developed copper complexes like Cu(Phe)2 proved to be the best at improving Cu status and its efficacy was further tested in a hypocupraemic herd, showing that the treated cattle maintained the serum copper level at an adequate level for at least 100 days. At the same time, analyses of copper, molybdenum, sulphur and phosphorous levels in grass were conducted throughout the year. The results showed that the copper content in grass was under adequate levels for most of the year, and the deficiency was more critical in autumn. Although the molybdenum and sulphur levels were low enough not to expect them to interfere with copper absorption, the Mo content may have been enhancing the copper deficiency [10].

### Arsenic in ground water

A thesis project and a multidisciplinary project have been carried out by environmental geologists and chemical toxicologists to look for Arsenic and other toxic metals in groundwater from the Raigon Aquifer in southwest Uruguay [11]. The groundwater quality has been the target of multiple studies in Uruguay. In particular, the Raigón Aquifer is of special interest because of its importance as a hydro-resource in the southern zone of Uruguay and for being responsible for a great part of the dairy farm water supply. Thus, little attention has been paid to the presence of toxic elements in the water. Preliminary research work done by this team has determined that arsenic levels were higher than 0.01 mg/L in water samples from this aquifer. This issue leads to the supposition that the population, as well as industrial and agricultural activities, could be consuming water with arsenic concentrations over the international maximum recommended limits of 0.01 mg/L. As it is fully described, these arsenic water levels may contribute to health problems over a long-term exposure [12]. The sampling campaign was done in fall 2007 and almost a hundred wells, with lithological profile available, were sampled with standard protocols, as representative of the aquifer.

The analysis were performed by ICP-MS and the results showed 80% samples with arsenic levels exceeding 0,01 mg/L ranging from 0,001 mg/L to an arsenic maximum concentration of 0.03 mg/L [13]. Therefore, this project is facing a typical interactive problem between geology and health (Medical Geology) and it requires a systematic evaluation of the toxicological interest, geological materials (chemical laboratory analyses) and the risks to the exposed population (human and animal ones) to characterize the water supply in connection with its toxic metal and metalloids contents. This research contributed to the assessment of exposure risks to human and animal populations, and eventually to the management and/or specific treatment of these resources to prevent long-term epidemiological problems.

In addition, since 2006, this arsenic Uruguayan working team, has been taking part in an Iberoamerican network project [14] regarding arsenic distribution, analytical methodologies development and remediation, and it is now developing feasible analytical methodologies for arsenic speciation.

### Training Courses and Future actions

Medical Geology is delivered as a curricular course to undergraduate students in the Faculty of Chemistry and updating training courses on Medical Geology have been carried out in Uruguay periodically since 2005 by one or all of the leaders, Drs Centeno, Finkelman and Selinus, to professionals, postgraduate students, bio scientists and geoscientists.

Future actions have also been planned to continue developing Medical Geology in this country. Uruguay hosted the 3rd Hemispherical Conference held in Montevideo, to promote interdisciplinary discussions and contributions from the geosciences and biosciences scientific communities to share the recent advances on environmental and health problems in this part of the world [15] and a project on a expert diploma with a similar program to those offered in other Universities, will be submitted to the University of the Republic’s authorities for consideration in the near future.

## 3. Final Considerations

In this presentation we updated the experience of Medical Geology in Uruguay, including some research studies that are being carried out. We emphasized the importance of the integration of the different University activities with the social and political actions for the management of all of these environmental health risk situations.

The main results of this new scientific approach in Uruguay are the development of feasible technologies, diagnosis tools, health impairment, and social inclusion. We conclude that Medical Geology is a growing field with multiple applications to solve local and regional health issues in Uruguay.
